# Equivalent efficacy study of QL1101 and bevacizumab on untreated advanced non-squamous non-small cell lung cancer patients: a phase 3 randomized, double-blind clinical trial

**DOI:** 10.20892/j.issn.2095-3941.2020.0212

**Published:** 2021-08-15

**Authors:** Tianqing Chu, Jun Lu, Minghong Bi, Helong Zhang, Wu Zhuang, Yan Yu, Jianhua Shi, Zhendong Chen, Xiaochun Zhang, Qisen Guo, Quan Liu, Huijuan Wu, Jian Fang, Yi Hu, Xiuwen Wang, Cuicui Han, Kai Li, Baohui Han

**Affiliations:** 1 Department of Pulmonary Medicine, Shanghai Chest Hospital, Shanghai Jiao Tong University, Shanghai 200030, China; 2 Department of Oncology, The First Affiliated Hospital of Bengbu Medical College, Bengbu 233004, China; 3 Department of Oncology, Tangdu Hospital, Air Force Medical University of PLA, Xi’an 710038, China; 4 Department of Medical Thoracic Oncology, Fujian Cancer Hospital, Fujian Medical University Cancer Hospital, Fuzhou 350014, China; 5 Department of Oncology, Harbin Medical University Cancer Hospital, Harbin 150081, China; 6 Department of Oncology, Linyi Cancer Hospital, Linyi 276000, China; 7 Department of Oncology, The Second Hospital of Anhui Medical University, Hefei 230601, China; 8 Department of Oncology, The Affiliated Hospital of Qingdao University, Qingdao 266071, China; 9 Department of Internal Medicine, Shandong Cancer Hospital Affiliated to Shandong University, Jinan 250117, China; 10 Department of Oncology, Affiliated Hospital of Jiang Nan University, Wuxi 214122, China; 11 Department of Internal Medicine, Henan Province Tumor Hospital, Zhengzhou University, Zhengzhou 450008, China; 12 Department of Thoracic Oncology II, Peking University Cancer Hospital and Institute, Beijing 100142, China; 13 Department of Oncology, Chinese PLA General Hospital, Beijing 100039, China; 14 Department of Oncology, Qilu Hospital of Shandong University, Jinan 250012, China; 15 Department of Clinical Research Centre, Qilu Pharmaceutical Co., Ltd, Jinan 250101, China; 16 Department of Thoracic Oncology, Tianjin Medical University Cancer Institute and Hospital, National Clinical Research Center for Cancer, Key Laboratory of Cancer Prevention and Therapy, Tianjin, Tianjin’s Clinical Research Center for Cancer, Tianjin 300060, China

**Keywords:** Biosimilar, bevacizumab, equivalence, non-squamous NSCLC, clinical efficacy

## Abstract

**Objective:**

This phase 3 study aimed to test equivalence in efficacy and safety for QL1101, a bevacizumab analogue in Chinese patients with untreated locally advanced non-squamous non-small cell lung cancer (NSCLC).

**Methods:**

Eligible patients were randomly assigned 1:1 to receive carboplatin and paclitaxel in combination with either QL1101 or bevacizumab, 15 mg/kg every 3-week for 6 cycles. This was followed by maintenance treatment with single agent QL1101 every 3-week. The primary end-point was objective response rate (ORR), with secondary end-points being progression-free survival (PFS), overall survival (OS), disease control rate (DCR), and adverse events (AEs).

**Results:**

Of 675 patients, 535 eligible patients were randomized to the QL1101 group (*n* = 269) and bevacizumab group (*n* = 266). ORRs were 52.8% and 56.8%, respectively, for the QL1101 and bevacizumab groups, with an ORR hazard ratio 0.93 (95% confidence interval: 0.8–0131.1). The PFS, OS, DCR, and AEs were comparable between the 2 groups, which remained the same after stratification according to epidermal growth factor receptor mutation or smoking history.

**Conclusions:**

QL1101 showed similar efficacy and safety profiles as compared to bevacizumab among Chinese patients with untreated locally advanced non-squamous NSCLC.

## Introduction

Bevacizumab, a recombinant humanized monoclonal antibody against vascular endothelial growth factor receptor, was approved by the United States Food and Drug Administration (FDA) in 2004 as a first-line treatment in combination with cytotoxic chemotherapy for patients with advanced colorectal cancer. Its clinical utility has been expanded to non-squamous non-small cell lung cancer (NSCLC) in 2006^1,2^. To date, bevacizumab is also approved in most countries (including the US, European Union, and China) for treatment of multiple cancers (NSCLC, colorectal cancer, and breast cancer, etc.)^1–3^. Financial burdens related to bevacizumab pose challenges to healthcare systems among many countries including China, so bevacizumab analogues are highly desirable to improve cost effectiveness^4–6^.

Multiple bevacizumab analogues (including ABP 215, QL1101, PF-06439535, and CT-P16, etc.) have been developed by different pharmaceutical companies^7–10^. ABP 215 was the first bevacizumab analogue approved by the US FDA in 2018^11^, while QL1101, another bevacizumab analogue, was approved by the National Medical Products Administration (NMPA) (China) in 2019. QL1101 was shown to have a similar structure, *in vitro* tumor growth inhibition, and pharmacokinetic profile as bevacizumab^8^. However, questions remain unanswered regarding the comparability in their efficacies and safety profiles. In this clinical trial (NCT03169335), we aimed to test the equivalence in efficacy and safety profiles for the bevacizumab analogue, QL1101, among Chinese patients with untreated local stage IIIb or stage IV non-squamous NSCLC.

## Materials and methods

### Study design

This study was a randomized, double-blind, multicenter, phase 3 clinical trial (ClinicalTrials.gov identifier: NCT03169335). Patients from 54 centers in China were enrolled between November 1, 2016 and July 31, 2018. The study was conducted according to the tenets of the Declaration of Helsinki, and was approved by the institutional independent ethics committee. Written informed consent was obtained from all participants before entering the trial.

### Patients and treatments

Of 675 patients screened for this clinical trial, 535 eligible patients were enrolled. The main inclusion criteria included: ages ≥ 18 years of age and < 75 years of age; pathologically- confirmed non-squamous stage IIIb or stage IV NSCLC; at least one measurable lesion per Response Evaluation Criteria in Solid Tumors, version 1.1 (RECIST 1.1); and an untreated or progressed disease and an Eastern Cooperative Oncology Group (ECOG) score of 0 or 1. Key exclusion criteria included squamous NSCLC (including mixed type adenocarcinoma squamous cell carcinoma); known sensitizing *EML4-ALK* translocations (patients without known status were permitted); known central nervous system metastases; major surgical resection 28 days prior to enrollment; and any prior systemic therapy (including chemotherapy, targeted therapy and immunotherapy). Patients were randomized to receive paclitaxel (175 mg/m^2^ Q21d IV) plus (carboplatin: AUC 6 Q21 IV) in combination with either QL1101 (15 mg/kg Q21d IV) or bevacizumab (15 mg/kg Q21d IV) for 4–6 cycles followed by maintenance therapy with QL1101(15 mg/kg Q21d IV).

### Randomization and masking

Patients were randomly assigned 1:1 to receive QL1101-based or bevacizumab-based treatment protocols with a block randomization scheme using a double-blind, computerized, and randomized list generator. The randomization factors included age (< 65 years or ≥ 65 years), sex (male or female), smoking history (yes or no), pathology [wild-type or epidermal growth factor receptor (EGFR) mutation], and ECOG (0 or 1). Packaging of the QL1101 and bevacizumab (supplied by Qilu Pharmaceutical Group) were identical and coded according to the random code list.

### Outcomes

The primary end-point was objective response rate (ORR) using an independent image blinding evaluation committee. ORR [including complete response (CR) and partial response (PR)] was determined using RESCIST 1.1 criteria. Secondary end-points were progression-free survival (PFS), overall survival (OS), and disease control rate (DCR; 6 months, 12 months, and 18 months, respectively). The safety end-point was used to compare treatment emergent adverse events (TEAEs) between the 2 groups.

### Statistical analysis

The primary end-point-ORR was calculated by using the approximate Gaussian distribution method, with the corresponding confidence interval set at 90%. Equivalence assessment was determined if the ORR ratio was 0.75–1.33. The equivalence assessment for secondary end-points (PFS, OS, and DCR) was performed using the Kaplan-Meier method where the Mantel-Cox test (including the chi-square and log-rank *P* value) were chosen. Equivalence evaluation in safety end-point was performed by comparing the incidence rates of adverse events (TEAE, serious TEAE, neutrophil reduction, and leukopenia) between the 2 groups.

## Results

**Figure 1:**
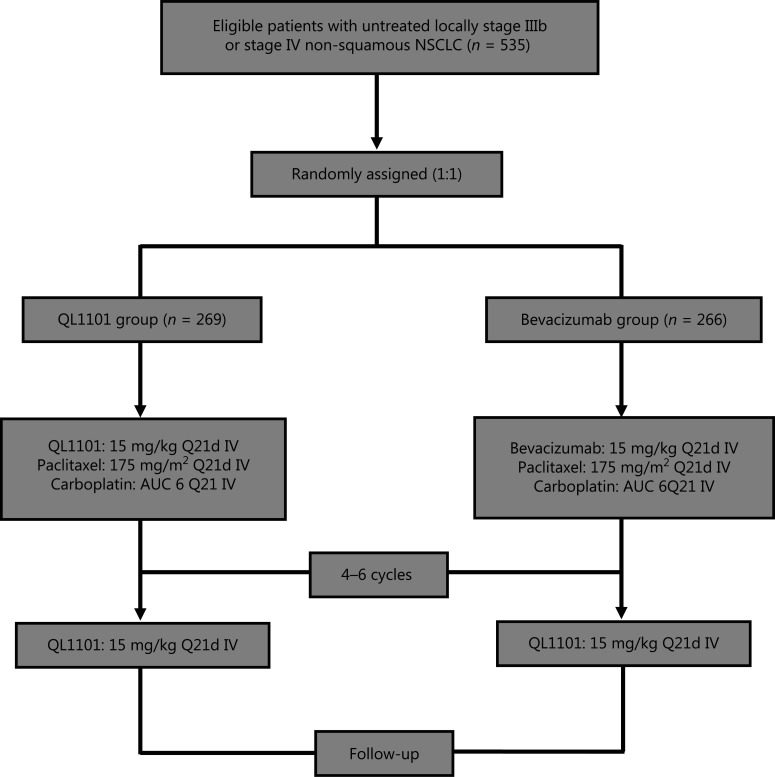
Study flowchart.

A total of 535 eligible patients with untreated locally stage IIIb or stage IV non-squamous NSCLC were enrolled and treatment assigned. All participants were randomized 1:1 to the QL1101 group [*n* = 269, 50.28%, of whom 158 (58.7%) were male, of whom 111 (41.3%) were female, with a median age of 59 (27–75) years of age] and the bevacizumab group [*n* = 266, 49.72%, of whom 160 (60.2%) were male, of whom 106 (39.8%) were female, with a median age of 58 (35–75) years of age] (**Figure 1**, **Supplementary Table S1**). Of the 535 patients, 104 patients (38.7%, QL1101 group) and 107 patients (40.2%, bevacizumab group) tested positive for a EGFR mutation; 125 patients (46.5%, QL1101 group) and 127 patients (47.7%, bevacizumab group) had a history of smoking, 26 patients (9.7%, QL1101 group), and 30 patients (11.3%, bevacizumab group) had a history of secondary malignancy. The baseline clinical characteristics are shown in **Supplementary Table S1**, and were well balanced between the 2 groups. All patients were followed-up to death, and the median follow-up durations were 14.7 (0.8–22.4) months for the QL1101 group and 15.2 (1.8–22.4) months for the bevacizumab group.

**Figure 2:**
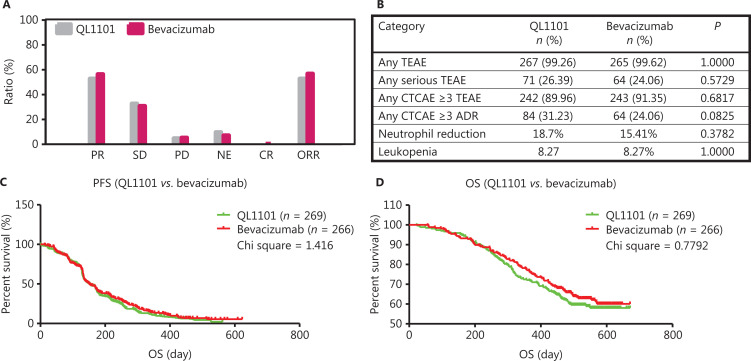
Equivalent therapeutic efficacy and safety were demonstrated between QL1101 and bevacizumab. (A) Similarity between QL1101 and bevacizumab was evaluated for the end-points of partial response, stable disease, progressive disease, not evaluated, complete response, and objective response rate. (B) Equivalence between QL1101 and bevacizumab was compared for safety including any treatment emergent adverse events (TEAEs), any serious TEAEs, any CTCAE ≥ 3 TEAE, any CTCAE ≥ 3 adverse drug response (ADR), neutrophil reduction, and leukopenia. (C, D) Similarities between QL1101 and bevacizumab were evaluated for the progression-free survival and overall survival end-points.

All 535 patients were included in the statistical analysis. At the time of data cutoff, 109 out of 269 patients (40.5%) in the QL1101 group died, while 99 out of 266 patients (37.2%) in the bevacizumab group died. Among patients receiving QL1101, 52.8% patients achieved partial response (PR) (142 patients), 32.7% stable disease (SD) (88 patients), 4.8% progressive disease (PD) (13 patients), 9.7% not evaluated (NE) (26 patients), and none with complete response (CR) (0 patients, 0%), ORR 52.8% (142 patients) were observed; while among patients in the bevacizumab group, PR (150 patients, 56.4%), SD (82 patients, 30.8%), PD (14 patients, 5.3%), NE (19 patients, 7.1%), CR (1 patient, 0.4%), and ORR 56.8% (151 patients) were observed (**Figure 2A**).

The median PFSs were 151 days for both the QL1101 group and the bevacizumab group [151 days, (HR = 1.112, 95% CI: 0.9334–1.326), chi-square = 1.416] (**Figure 2C**). The survivals at 6 months, 12 months, and 18 months were 92.9% (250 of 269 patients), 72.1% (194 of 269 patients), and 28.3% (76 of 269 patients), respectively, in the QL1101 group, as compared to 91.8% (247 of 266 patients), 75.8% (204 of 266 patients), and 33.5% (76 of 266 patients) for the bevacizumab group, respectively. The median OSs were not reached [HR = 1.130, 95% confidence interval (CI): 0.8611–1.484, chi-square = 0.7792] (**Figure 2D**). Either QL1101-induced or bevacizumab- induced AEs were nearly equivalent, except the evaluation of any *Common Terminology Criteria for Adverse Events* (CTCAE) ≥ 3 adverse drug reactions (ADR) [QL1101: 84 patients (31.23%) *vs.* bevacizumab: 64 patients (24.06%), *P* = 0.0825] (**Figure 2B**).

Subgroup analyses were conducted according to age, sex, smoking history, pathology, tumor history, and ECOG; there was no statically significant difference found for the PFS. Regarding the OS (**Table 1**) among patients with a EGFR mutation, the median OS of the bevacizumab group was longer than the median OS of the QL1101 group [QL1101 (*n* = 104) *vs.* bevacizumab (*n* = 107): HR = 1.617 (0.9382–2.788), chi-square = 2.994, log-rank *P* value = 0.0835] (**Supplementary Figure S1A**). A similar result was found in the other 2 subgroups. As shown in **Supplementary Figure S1B**, patients with a smoking history receiving QL1101 therapy showed a lower OS than those patients receiving bevacizumab therapy [QL1101 (*n* = 66) *vs.* bevacizumab (*n* = 72): HR = 1.610 (0.9893–2.619), chi-square = 3.674, log-rank *P* value = 0.0553]. Regarding analysis of the ECOG 0 subgroup, the results were between those of the EGFR mutation subgroup and ever smoker subgroup [QL1101 (*n* = 61) *vs.* bevacizumab (*n* = 65): HR = 1.700 (0.9502–3.040), chi-square = 3.196, log-rank *P* value = 0.0738)] (**Supplementary Figure S1C**). These results indicated that there was little difference in therapeutic efficiencies between QL1101 and bevacizumab treatments of some subgroups of patients with untreated local stage IIIb or stage IV non-squamous NSCLC.

**Table 1:** Equivalent therapeutic efficacy between QL1101 and bevacizumab was evaluated for the subgroups of baseline characteristics

	PFS (days)				OS (days)			
	QL1101^a^	Bevacizumab^b^	HR (95% CI)^c^	*P* ^d^	QL1101^a^	Bevacizumab^b^	HR (95% CI)^c^	*P* ^d^
Age (years)								
< 65	168	161	1.055 (0.8632–1.290)	0.2749	Undefined	Undefined	1.096 (0.7898–1.522	0.3025
≥ 65	132	140	1.311 (0.9113–1.885)	2.128	504	501	1.034 (0.6868–1.557)	0.0256
Gender								
Male	153	151	1.109 (0.8834–1.392)	0.7947	475	Undefined	1.288 (0.9342–1.775)	2.384
Female	141	150.1	1.138 (0.8627–1.502)	0.8381	Undefined	Undefined	0.880 (0.5257–1.473)	0.2364
Smoking history								
Never	152	168	1.121 (0.8803–1.429)	0.8611	Undefined	Undefined	1.050 (0.6841–1.610)	0.0491
Ever	164	139	1.173 (0.8267–1.665)	0.8001	462	Undefined	1.610 (0.9893–2.619)	3.674
Still	136	134	0.9681 (0.6633–1.413)	0.0283	Undefined	466	0.8480 (0.5057–1.422)	0.3905
Pathology								
Wild type	150	144	1.058 (0.8448–1.325)	0.2409	Undefined	566	1.003 (0.7329–1.374)	0.0275
EGFR mutation	151.5	169	1.188 (0.8967–1.573)	1.44	Undefined	Undefined	1.617 (0.9382–2.788)	2.994
Tumor history								
Yes	152	177	1.281 (0.7342–1.439)	0.7609	Undefined	Undefined	0.651 (0.2440–1.736)	0.7354
No	151	148.5	1.089 (0.9050–1.311)	0.8161	Undefined	Undefined	1.175 (0.8854–1.560)	1.249
ECOG								
0	155	132	0.8692 (0.6022–1.255)	0.5599	Undefined	Undefined	1.700 (0.9502–3.040)	3.196
1	151	170	1.192 (0.9752–1.456)	2.938	Undefined	Undefined	1.010 (0.7423–1.375)	0.004

^a^The bevacizumab analogue was sourced from Qilu Pharmaceutical Co., Ltd, China. ^b^Bevacizumab was sourced from Roche, China. ^c^Hazard ratio (HR) [95% confidence interval (CI)]. ^d^Chi-square value using the Mantel-Cox test. PFS, progression free survival; OS, overall survival; ECOG, Eastern Cooperative Oncology Group; EGFR, epidermal growth factor receptor.

**Figure 3:**
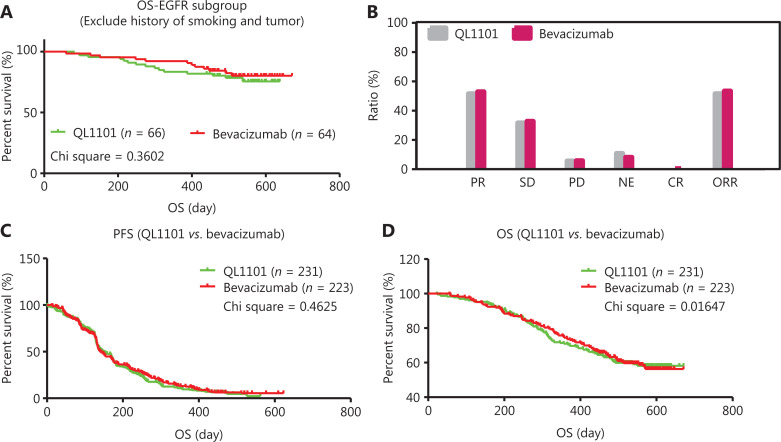
Evaluation of equivalent therapeutic efficacy after excluding interference factors. (A) Equivalent overall survival (OS) evaluation in EGFR-mutated patients, excluding patients with a history of smoking and tumors. (B) After excluding the interference factors, better equivalence between QL1101 and bevacizumab was observed for the end-points of partial response, stable disease, progressive disease, not evaluated, complete response, and objective response rate. (C, D) After excluding interference factors, better equivalence between QL1101 and bevacizumab was found for the end-points of progression-free survival and overall survival.

To understand how the differences originated, we performed further analyses on the clinical data. Patients with EGFR mutations in the ECOG 0 subgroup received more OS benefit when they were treated with bevacizumab (*n* = 24) rather than QL1101 (*n* = 20) (log-rank *P* value = 0.0047) (**Supplementary Figure S2A**). Regarding the EGFR mutation patients in the ever smoking history subgroup, there was slightly more OS benefit after bevacizumab therapy (*n* = 19) when compared with QL1101 therapy (*n* = 17) (log-rank *P* value = 0.1070) (**Supplementary Figure S2B**). In these patients, we first assumed that the cause of the therapeutic efficiency difference may have been attributed to some patients in the EGFR mutation subgroup. We then analyzed the equivalent efficacy of those patients with EGFR mutations after excluding patients with a history of smoking and tumors. The results showed that the therapeutic efficacy between QL1101 therapy and bevacizumab therapy was much more similar (chi-square = 0.3602) (**Figure 3A**).

After eliminating imbalanced factors, we further analyzed the response for all patients (*n* = 454) between the QL1101 (*n* = 231) and bevacizumab groups (*n* = 223). The responsive score including the PR (119 patients, 51.5%), SD (73 patients, 31.6%), PD (13 patients, 5.6%), NE (25 patients, 10.8%), CR (0 patients, 0%), and ORR (119 patients, 52.8%) were defined after patients received QL1101 therapy, and the PR (118 patients, 52.9%), SD (73 patients, 32.7%), PD (13 patients, 5.8%), NE (18 patients, 8.1%), CR (1 patients, 0.4%), and ORR (119 patients, 53.4%) were defined after patients received bevacizumab therapy (**Figure 3B**). The therapeutic efficacy between QL1101 and bevacizumab showed more equivalence, with remarkable equivalence after OS analysis (chi-square = 0.01647) (**Figure 3C, 3D**). Analyses of the equivalence of different subgroups showed that nearly all subgroups had much more similar therapeutic efficacies, either using PFS analysis or OS analysis (**Table 2**). Finally, we conducted responsive analyses on patients who received QL1101 or bevacizumab; the results suggested that nearly all subgroups (including age, sex, smoking history, pathology, and tumor history) showed no difference, while after PFS analyses, patients with age < 65 years, female, no smoking history, and EGFR mutations, received more OS benefit than patients with age ≥ 65 years, male, smoking history, and no EGFR mutation (**Supplementary Table S2**). These results indicated that some baseline characteristics were associated with OS outcomes of patients with untreated local stage IIIb or stage IV non-squamous NSCLC, which did not affect the therapeutic efficacy while being treated with bevacizumab or QL1101 as the first-line therapy.

**Table 2:** Equivalent therapeutic efficacy between QL1101 and bevacizumab was evaluated for the subgroups after excluding the interference factors.

	PFS (days)				OS (days)			
	QL1101^a^	Bevacizumab^b^	HR (95% CI)^c^	*P* ^d^	QL1101^a^	Bevacizumab^b^	HR (95% CI)^c^	*P* ^d^
Age (years)								
< 65	169	147	0.9985 (0.8014–1.244)	< 0.001	Undefined	Undefined	0.9792 (0.6854–1.399	0.0134
≥ 65	131	137	1.288 (0.8777–1.890)	1.673	459	508	1.150 (0.6985–1.894)	0.3025
Gender								
Male	150	134	1.065 (0.825–1.374)	0.2321	459	566	1.161 (0.8203–1.643)	0.7084
Female	141	147	1.093 (0.8203–1.456)	0.3687	Undefined	Undefined	0.8216 (0.4839–1.395)	0.5290
Smoking history								
Never	150	168	1.130 (0.8807–1.450)	0.9231	Undefined	Undefined	1.032 (0.6695–1.592)	0.0209
Ever	170	133	0.9533 (0.6372–1.426)	0.0541	459	497	1.004 (0.6791–1.484)	< 0.001
Still	130	121	0.9171 (0.5900–1.426)	0.1479	Undefined	396.5	0.6473 (0.3583–1.169)	2.078
Pathology								
Wild type	150	144	1.058 (0.8448–1.325)	0.2409	Undefined	566	1.003 (0.7329–1.374)	0.0275
EGFR mutation	142.5	138	1.091 (0.7627–1.561)	0.2286	Undefined	Undefined	1.260 (0.5921–2.682)	0.3602
Tumor history								
Yes	146	175	1.602 (0.7613–3.371)	1.541	Undefined	513	0.3735 (0.1200–1.163)	2.888
No	145	140	1.033 (0.8479–1.259)	0.8161	Undefined	Undefined	1.085 (0.8038–1.465)	0.2838
ECOG								
0	158	132	0.7639 (0.5121–1.139)	1.744	Undefined	Undefined	1.281 (0.6763–2.426)	0.5772
1	144	154	1.174 (0.9451–1.459)	2.105	Undefined	Undefined	0.9589 (0.6922–1.328)	0.0638

^a^Bevacizumab biosimilar sourced from Qilu Pharmaceutical Co., Ltd, China. ^b^Bevacizumab sourced from Roche China. ^c^Hazard Ratio (95% CI of ratio. ^d^Chi square value using Mantel-Cox test.

## Discussion

Lung cancer is the leading cause of cancer death worldwide, with NSCLC accounting for approximately 85% of all cases^12,13^. Anti-angiogenesis drugs play an important role in preventing disease progression^2,14–16^. Since US FDA approval of bevacizumab in treating non-squamous NSCLC in October 2006, access to bevacizumab has been limited because of its high cost^1,4–6^, which makes bevacizumab analogues highly desirable^5^. Bevacizumab analogue, QL1101, was approved by the NMPA (China) in November 2019. This study therefore aimed to assess the possible equivalence in efficacy and toxicity of the bevacizumab analogue, QL1101, among Chinese patients with untreated local stage IIIb or stage IV non-squamous NSCLC.

Published data has shown comparable efficacies and safety profiles for bevacizumab and its ABP125 and PF-06439535 analogues among patients with non-squamous NSCLC^7,9^. Patients enrolled in these two clinical trials were mostly from Europe and the USA, and patients with recurrent disease after adjuvant or neoadjuvant treatment were included in these trials. The present study focused on the Chinese population who were systemic treatment naïve. Similar to the ABP125 study, patients with confirmed EGFR mutations were included in this trial, which also served as a stratification parameter.

In this phase 3 clinical study, the primary end-point (ORR) and secondary end-points (including PFS, OS, DCR, and safety) were similar between the analogue and bevacizumab groups. ORR has been an important parameter in many biosimilar studies^7,9,17,18^.

Because of EGFR-tyrosine kinase inhibitor (TKI) treatments, studies have shown that NSCLC patients with EGFR mutations received more OS benefit than those without EGFR mutations^19^. Moreover, patients with a smoking history had decreased OS, when compared with patients without a smoking history^20^. These factors affecting OS analysis have been considered before double-blind randomization, and have been incorporated into the groupings. We found that 3 subgroups (EGFR mutation, ever smoking history, and ECOG 0) had a difference in OS between QL1101 and bevacizumab therapies, indicating the presence of an imbalance factor between the QL1101 and bevacizumab groups. After analysis, we found differences between the 2 groups were attributed to EGFR mutation patients with a history of smoking or tumors. After excluding interference factors, we then found a significant equivalence for primary end-points (ORR) and secondary end-points (OS), and also found good equivalence in subgroup analyses. These results indicated that we should fully consider basic baseline characteristics, which were more objective in showing a difference in comparative clinical trials for other anti-cancer analogues.

## Conclusions

This study showed equivalences in efficacy (including primary end-point ORR, and secondary end-point PFS, OS, and DCR) and safety profiles when comparing bevacizumab analogue, QL1101, with bevacizumab among patients with non-squamous NSCLC in China.

## References

[1] Hurwitz H, Fehrenbacher L, Novotny W, Cartwright T, Hainsworth J, Heim W (2004). Bevacizumab plus irinotecan, fluorouracil, and leucovorin for metastatic colorectal cancer. N Engl J Med.

[2] Sandler A, Gray R, Perry MC, Brahmer J, Schiller JH, Dowlati A (2006). Paclitaxel-carboplatin alone or with bevacizumab for non-small-cell lung cancer. N Engl J Med.

[3] Cameron D, Brown J, Dent R, Jackisch C, Mackey J, Pivot X (2013). Adjuvant bevacizumab-containing therapy in triple-negative breast cancer (BEATRICE): primary results of a randomised, phase 3 trial. Lancet Oncol.

[4] Huang HY, Wu DW, Ma F, Liu ZL, Shi JF, Chen X (2020). Availability of anticancer biosimilars in 40 countries. Lancet Oncol.

[5] Lyman GH, Zon R, Harvey RD, Schilsky RL (2018). Rationale, opportunities, and reality of biosimilar medications. N Engl J Med.

[6] Santos SB, Lobo JMS, Silva AC (2019). Biosimilar medicines used for cancer therapy in Europe: a review. Drug Discov Today.

[7] Thatcher N, Goldschmidt JH, Thomas M, Schenker M, Pan Z, Rodriguez LPA (2019). Efficacy and safety of the biosimilar ABP 215 compared with bevacizumab in patients with advanced nonsquamous non-small cell lung cancer (MAPLE): a randomized, double-blind, phase III study. Clin Cancer Res.

[8] Liu YN, Huang J, Guo C, Yang S, Ye L, Wu S (2020). A randomized, double-blind, single-dose study to evaluate the biosimilarity of QL1101 with bevacizumab in healthy male subjects. Cancer Chemoth Pharmacol.

[9] Reinmuth N, Bryl M, Bondarenko I, Syrigos K, Vladimirov V, Zereu M (2019). PF-06439535 (a bevacizumab biosimilar) compared with reference bevacizumab (Avastin®), both plus paclitaxel and carboplatin, as first-line treatment for advanced non-squamous non-small-cell lung cancer: a randomized, double-blind study. Biodrugs.

[10] Cho SH, Han S, Ghim JL, Nam MS, Yu S, Park T (2019). A randomized, double-blind trial comparing the pharmacokinetics of CT-P16, a candidate bevacizumab biosimilar, with its reference product in healthy adult males. Biodrugs.

[11] Casak SJ, Lemery SJ, Chung J, Fuchs C, Schrieber SJ, Chow EC (2018). FDA’s approval of the first biosimilar to bevacizumab. Clin Cancer Res.

[12] Jemal A, Bray F, Center MM, Ferlay J, Ward E, Forman D (2011). Global cancer statistics. CA Cancer J Clin.

[13] Siegel RL, Miller KD, Jemal A (2018). Cancer statistics, 2018. CA Cancer J Clin.

[14] Han BH, Li K, Wang QM, Zhang L, Shi J, Wang Z (2018). Effect of anlotinib as a third-line or further treatment on overall survival of patients with advanced non-small cell lung cancer The ALTER 0303 phase 3 randomized clinical trial. JAMA Oncol.

[15] Lu J, Zhong H, Chu TQ, Zhang X, Li R, Sun J (2019). Role of anlotinib-induced CCL2 decrease in antiangiogenesis and response prediction for nonsmall cell lung cancer therapy. Eur Respir J.

[16] Lu J, Zhong H, Wu J, Chu T, Zhang L, Li H (2019). Circulating DNA-based sequencing guided anlotinib therapy in non-small cell lung cancer. Adv Sci.

[17] Rugo HS, Barve A, Waller CF, Hernandez-Bronchud M, Herson J, Yuan J (2017). Effect of a proposed trastuzumab biosimilar compared with trastuzumab on overall response rate in patients with ERBB2 (HER2)-positive metastatic breast cancer: a randomized clinical trial. JAMA.

[18] Jurczak W, Moreira I, Kanakasetty GB, Munhoz E, Echeveste MA, Giri P (2017). Rituximab biosimilar and reference rituximab in patients with previously untreated advanced follicular lymphoma (ASSIST-FL): primary results from a confirmatory phase 3, double-blind, randomised, controlled study. Lancet Haematol.

[19] Zhou C, Wu Y, Chen G, Feng J, Liu XQ, Wang C (2015). Final overall survival results from a randomised, phase III study of erlotinib versus chemotherapy as first-line treatment of EGFR mutation-positive advanced non-small-cell lung cancer (OPTIMAL, CTONG-0802). Ann Oncol.

[20] Dogan S, Shen R, Ang DC, Johnson ML, D’Angelo SP, Paik PK (2012). Molecular epidemiology of EGFR and KRAS mutations in 3,026 lung adenocarcinomas: higher susceptibility of women to smoking-related KRAS-mutant cancers. Clin Cancer Res.

