# Supplementary material for: Equivalent efficacy study of QL1101 and bevacizumab on untreated advanced non-squamous non-small cell lung cancer patients: a phase 3 randomized, double-blind clinical trial
Source: Cancer Biol Med. 2021 Aug 15;18(3):816–24. doi: 10.20892/j.issn.2095-3941.2020.0212 (PMC8330542; doi:10.20892/j.issn.2095-3941.2020.0212)
Supplement: Supplementary file 1 [file cbm-18-816-s001.pdf]

Supplementary materials

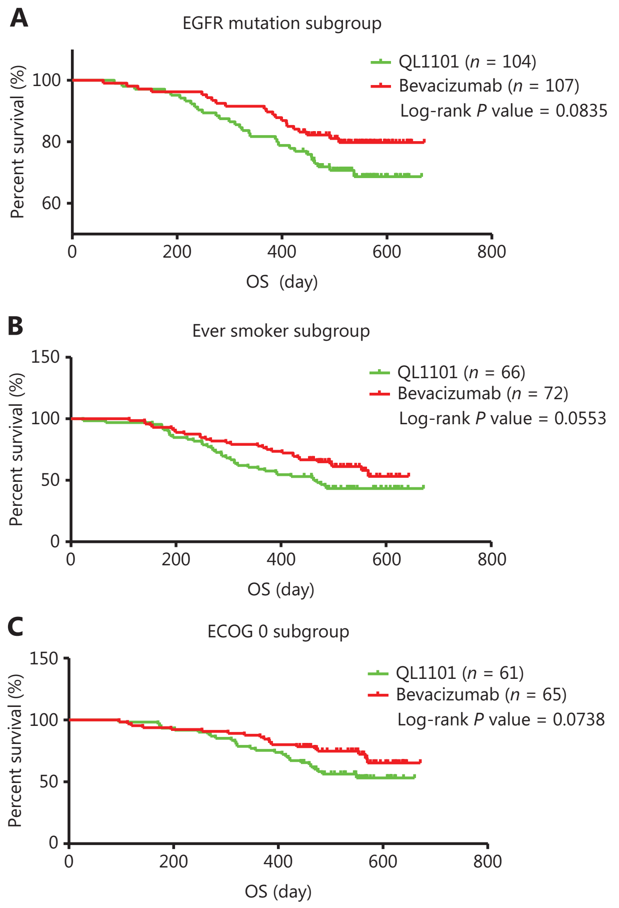

Figure S1 Differences of overall survival (OS) outcomes between QL1101 and bevacizumab were found for the subgroups of epidermal growth factor receptor (EGFR) mutation, ever smoker, and Eastern Cooperative Oncology Group (ECOG) 0. (A–C). The patients (including the EGFR mutation, ever smoker, and ECOG 0 subgroups) received a slightly longer OS benefit after bevacizumab therapy than those after QL1101 therapy.

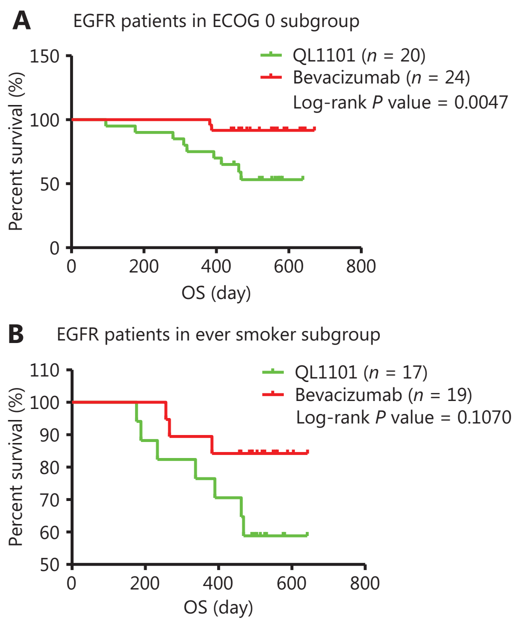

Figure S2 (A) Epidermal growth factor receptor (EGFR)-mutated patients in the Eastern Cooperative Oncology Group (ECOG) 0 subgroup received significant overall survival (OS) benefit from bevacizumab than those who were treated with QL1101. (B) EGFR-mutated patients in the ever smoker subgroup received slightly more OS benefit from bevacizumab than those from QL1101.

Table S1 Patient demographics and baseline characteristics

Characteristics	QL1101[a] (*n* = 269)	Bevacizumab[b] (*n* = 266)
No. of patients (%)		
Age (years)		
Median (range)	59 (27–75)	58 (35–75)
< 65	206 (76.6)	201 (76.6)
≥ 65	63 (23.4)	65 (23.4)
Gender		
Male	158 (58.7)	160 (60.2)
Female	111 (41.3)	106 (39.8)
Smoking history		
Never	144 (53.5)	139 (52.3)
Ever	66 (24.5)	72 (27.1)
Still	59 (22.0)	55 (20.6)
Pathology		
Wild type	165 (61.3)	159 (59.8)
EGFR mutation	104 (38.7)	107 (40.2)
Tumor history		
Yes	26 (9.7)	30 (11.3)
No	243 (90.3)	236 (88.7)
ECOG		
0	61 (22.7)	65 (24.4)
1	208 (77.3)	201 (75.6)
BMI	22.6 (14.8–33.7)	22.8 (13.5–31.3)

[a]Bevacizumab biosimilar sourced from Qilu Pharmaceutical Co., Ltd, China. [b]Bevacizumab sourced from Roche China. BMI, body mass index; EGFR, epidermal growth factor receptor; ECOG, Eastern Cooperative Oncology Group.

Table S2 Differences in PFS and OS outcomes among the subgroups after bevacizumab therapy as the first-line therapy

	PFS (days)		OS (days)	
	QL1101 or Bevacizumab	*P*[a]	QL1101 or Bevacizumab	*P*[a]
Age (years)				
< 65 (*n* = 340)	152	0.2250	Undefined	0.0002
≥ 65 (*n* = 114)	132		486	
Gender				
Male (*n* = 252)	142	0.1119	497	< 0.0001
Female (*n* = 202)	144		Undefined	
Smoking history				
No (*n* = 266)	152	0.0601	Undefined	< 0.0001
Yes (*n* = 188)	134		475	
Pathology				
Wild type (*n* = 324)	145	0.2337	566	< 0.0001
EGFR mutation (*n* = 130)	141		Undefined	
Tumor history				
Yes (*n* = 34)	148	0.5413	Undefined	0.6534
No (*n* = 420)	144		Undefined	
ECOG				
0 (*n* = 106)	138.5	0.0139	Undefined	0.1674
1 (*n* = 348)	149.5		Undefined	

[a]Log-rank *P* value using the Mantel-Cox test. EGFR, epidermal growth factor receptor; ECOG, Eastern Cooperative Oncology Group; OS, overall survival; PFS, progression free survival.
